# Effect of constraint-induced movement therapy on lower extremity motor dysfunction in post-stroke patients: A systematic review and meta-analysis

**DOI:** 10.3389/fneur.2022.1028206

**Published:** 2022-11-21

**Authors:** Mingze Zhou, Yang Tu, Jiarui Cui, Ping Gao, Ting Yi, Jun Wang, Qinghong Hao, Hui Li, Tianmin Zhu

**Affiliations:** ^1^School of Health Preservation and Rehabilitation, Chengdu University of Traditional Chinese Medicine, Chengdu, China; ^2^School of Preclinical Medicine, Chengdu University, Chengdu, China

**Keywords:** constraint-induced movement therapy, post-stroke, lower extremity, motor dysfunction, meta-analysis

## Abstract

**Objective:**

Constraint-induced movement therapy (CIMT) is a common treatment for upper extremity motor dysfunction after a stroke. However, whether it can effectively improve lower extremity motor function in stroke patients remains controversial. This systematic review comprehensively studies the current evidence and evaluates the effectiveness of CIMT in the treatment of post-stroke lower extremity motor dysfunction.

**Methods:**

We comprehensively searched randomized controlled trials related to this study in eight electronic databases (PubMed, Embase, The Cochrane Library, Web of Science, CBM, CNKI, WAN FANG, and VIP). We evaluated CIMT effectiveness against post-stroke lower extremity motor dysfunction based on the mean difference and corresponding 95% confidence interval (95% CI). We assessed methodological quality based on the Cochrane Bias Risk Assessment Tool. After extracting the general information, mean, and standard deviation of the included studies, we conducted a meta-analysis using RevMan 5.3 and Stata 16.0. The primary indicator was the Fugl-Meyer Assessment scale on lower limbs (FMA-L). The secondary indicators were the Berg balance scale (BBS), 10-meter walk test (10MWT), gait speed (GS), 6-min walk test (6MWT), functional ambulation category scale (FAC), timed up and go test (TUGT), Brunnstrom stage of lower limb function, weight-bearing, modified Barthel index (MBI), functional independence measure (FIM), stroke-specific quality of life questionnaire (SSQOL), World Health Organization quality of life assessment (WHOQOL), and National Institute of Health stroke scale (NIHSS).

**Results:**

We initially identified 343 relevant studies. Among them, 34 (totaling 2,008 patients) met the inclusion criteria. We found that patients treated with CIMT had significantly better primary indicator (FMA-L) scores than those not treated with CIMT. The mean differences were 3.46 (95% CI 2.74–4.17, P < 0.01, I2 = 40%) between CIMT-treated and conventional physiotherapy-treated patients, 3.83 (95% CI 2.89–4.77, P < 0.01, I2 = 54%) between patients treated with CIMT plus conventional physiotherapy and patients treated only with conventional physiotherapy, and 3.50 (95% CI 1.08–5.92, P < 0.01) between patients treated with CIMT plus western medicine therapy and those treated only with western medicine therapy. The secondary indicators followed the same trend. The subgroup analysis showed that lower extremity CIMT with device seemed to yield a higher mean difference in FMA-L scores than lower extremity CIMT without device (4.52, 95% CI = 3.65–5.38, *P* < 0.01 and 3.37, 95% CI = 2.95–3.79, *P* < 0.01, respectively).

**Conclusion:**

CIMT effectively improves lower extremity motor dysfunction in post-stroke patients; however, the eligible studies were highly heterogeneous.

**Systematic review registration**: https://www.crd.york.ac.uk/prospero/display_record.php?RecordID=277466.

## Introduction

Stroke has become a global health concern due to its extremely high mortality and disability rates. More than 20 million people suffer from stroke yearly, and only three-quarters survive (1). Limb motor dysfunction caused by stroke impairs patients' ability to live independently and their quality of life. Furthermore, patients may require long-term care, which burdens families and communities (2). Hemiplegia is a paralysis on one side of the body caused by pyramidal tract lesions and is a common post-stroke pathological manifestation. One of its main characteristics is the asymmetric motor pattern resulting from an excessive use of the unimpaired limb (3). Such a dependence can enhance the incoming information of the sensorimotor cortex and inhibit the use of the impaired limb, resulting in asymmetric and spasmodic patterns that complicate daily life (4).

Stroke can dramatically affect lower limb movement, resulting in abnormal movement patterns (5–7). Two-thirds of patients need help to walk 6 months after the stroke, and half cannot complete a 6-min walk test 1 year after the stroke (8–10). After effective rehabilitation, only one-third of patients return to work in the first year (11, 12). Therefore, it is necessary to help stroke patients recover lower limb motor function to enhance their independent living ability and allow them to rejoin society as soon as possible.

Constraint-induced movement therapy (CIMT), a motor rehabilitation technique, is often used to restore impaired limb motor function after a stroke to help bring back activities of daily living and reduce learned nonuse (13). The substantial positive results obtained with the upper extremity protocol have led to the development of CIMT for the lower extremity, an intervention to improve lower extremity function. Traditional CIMT involves a long period of limb limitation and intensive training, increasing patients' physical and mental pain (14). Unlike the traditional CIMT, the modified CIMT appropriately reduces the impaired limb's training intensity and the unimpaired limb's restriction time (15, 16), increasing the protocol's applicability, especially for older patients.

The lower extremity CIMT protocol includes (1) intensive practice of the functional activities, (2) limiting reliance on the unimpaired lower limb, (3) transfer of the gains from the training session to the family or community rehabilitation with a “transfer package” and (4) strong encouragement to use the impaired lower limb with improved coordination (17). However, unlike upper extremity CIMT, which uses a substantial constraint on the unimpaired lower limb (e.g., a padded mitt), the “constraint” for lower extremity CIMT can be behavioral, physical, or both (18). To stimulate walking ability and to overcome general inactivity, lower extremity CIMT consists of massed or repetitive practice of lower limb tasks (e.g., treadmill walking, over-ground walking, sit-to-stand, lie-to-sit, step climbing, and various balance and support exercises) (19). CIMT can improve gait parameters such as walking ability and gait speed, as well as the quantity and quality of movement (20–24).

A previous meta-analysis including six British randomized controlled trials (RCTs) of lower extremity CIMT intervention in stroke patients found that different CIMT protocols improved lower limb function (25). Silva and co-workers (26) observed that 2 weeks of CIMT (with the unimpaired ankle bearing a weight) did not improve lower limb motor function. Uswatte's study (27) also reported that upper limb restraint played a less critical role in CIMT benefits. Because gait training after stroke involves both the unimpaired and impaired legs, some studies (28, 29) do not recommend restricting the unimpaired leg with devices, as they can substantially alter leg inertia or normal gait patterns. Therefore, this systematic review and meta-analysis aimed to determine the effectiveness of CIMT on impaired lower limb function after stroke and to evaluate whether devices affect CIMT effectiveness.

## Methods

The protocol of this systematic review and meta-analysis was registered in PROSPERO (CRD42021277466).

### Search strategy

A systematic literature search was conducted in eight databases: PubMed, EMBASE, the Cochrane Library, Web of Science, China Biology Medicine (CBM), China National Knowledge Infrastructure (CNKI), Wan Fang Data, and the Chinese Science and Technology Periodical Database (VIP) from initiation up to December 2021. All the studies were limited to English and Chinese. All searches were based on the following keywords: “constraint induced movement therapy”, “stroke”, “lower extremity”, and “randomized controlled trial”. The complete search strategy of WOS is shown in Supplementary Figure S1.

### Inclusion criteria

We included studies meeting the following criteria: (1) studies on patients with motor impairment of the lower limbs after a stroke (regardless of stroke type, duration, affected brain area, or hemiplegia side); (2) studies that evaluated CIMT, modified CIMT, and any interventions conforming to the lower extremity CIMT core strategy (intensive practice of the functional activities and restraint of the less-affected lower extremity by the device or behavioral procedures), regardless of the treatment's frequency, duration and length; (3) studies that were RCTs; (4) studies written in English or Chinese.

### Exclusion criteria

We excluded studies that: (1) did not obtain results of interest; (2) were one of the following types: opinions, case reports, case series, conference papers, editorials, abstracts, or crossover studies; (3) were unavailable.

### Outcome measures

The main outcome measure was the Fugl-Meyer Assessment scale on lower limbs (FMA-L), which is commonly used to assess lower extremity motor function. Secondary outcomes included the Berg balance scale (BBS), 10-meter walk test (10MWT), gait speed (GS), 6-min walk test (6MWT), functional ambulation category scale (FAC), timed up and go test (TUGT), Brunnstrom stage of lower limb function, weight-bearing, modified Barthel index (MBI), functional independence measure (FIM), stroke-specific quality of life questionnaire (SSQOL), World Health Organization quality of life assessment (WHOQOL), and National Institute of Health stroke scale (NIHSS).

### Data extraction

We first imported the retrieved literature into EndNote X9 and automatically removed duplicate entries. Two reviewers (MZ and PG) screened the papers' titles and abstracts according to predefined inclusion and exclusion criteria. Then, two examiners reviewed the remaining full texts separately. They collected the following information: first author's name, year of publication, origin country, subjects' ethnicity, study design, sample size, mean age, stroke duration, grading criteria, interventions (including intensity and duration) in the experimental and control groups, and scores on the scale of interest (mean scores and standard deviation). Disagreements were resolved by consensus discussion between two authors (MZ and PG) or by consulting a third reviewer (YT).

### Quality assessment

Two reviewers (MZ and YT) independently assessed the methodological quality of the included studies using the Cochrane Bias Risk Assessment Tool. It consists in rating the risk of bias of seven items (as “low”, “unclear”, or “high”). Next, we rated studies with seven low-risk items as “high-quality” and those with one or more high-risk or unclear items as “low-quality”.

### Data analysis

We performed all statistical analyses using RevMan 5.3 and Stata 16.0. Because all variables included in the study were continuous, we analyzed the magnitude of the effects using the mean difference (MD) or standard MD and 95% confidence interval (CI). We also estimated the interstudy heterogeneity using the chi-squared test and the I^2^ statistic. A *P* < 0.05 or an I^2^ statistic > 50% indicated unaccepted variability among the included studies, and a random-effects model would be used to analyze the data. To explore the source of the heterogeneity, we performed sensitivity analyses. We estimated publication bias by constructing a funnel plot of each trial's MD and the standard error (Supplementary material). Next, we assessed the funnel plot asymmetry using Egger's test. Finally, we considered that *P* < 0.05 indicated significant publication bias.

## Results

### Literature search

**Figure 1:**
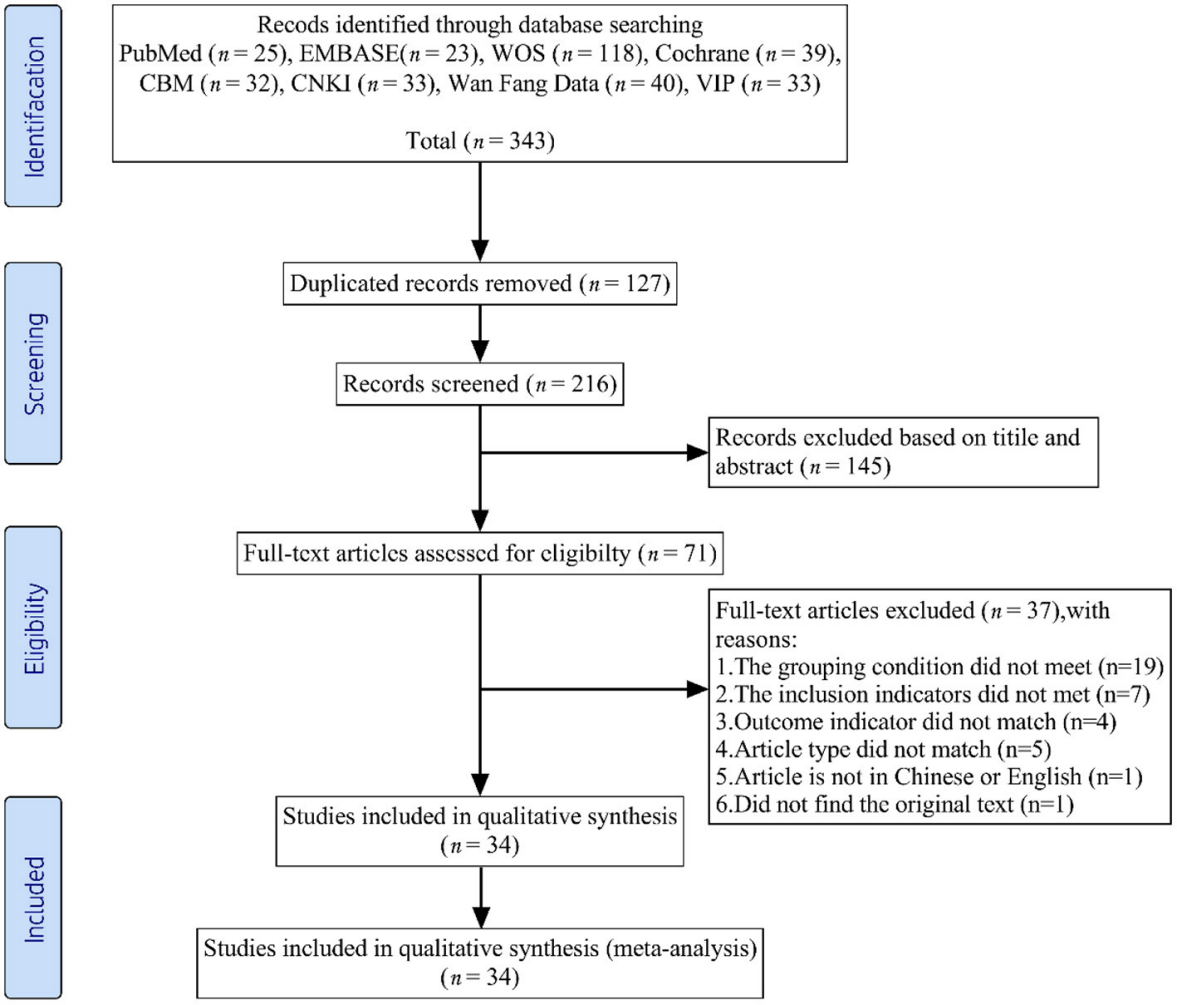
Flow diagram of the study inclusion and exclusion process.

We initially extracted 343 records from the eight databases. After the first scanning stage, we removed 127 duplicates. Of the remaining 216 records, we excluded 182 articles that did not meet the predefined inclusion criteria. Ultimately, this systematic review and meta-analysis included 34 RCTs (26, 30–62). The literature selection process appears in Figure 1.

### Main characteristics

Table 1 lists the main characteristics of the included studies. This meta-analysis included 34 studies with a total of 2008 participants (1,003 in the experimental group and 1,005 in the control group). All the articles were published between 2008 and 2021. The experimental group in these studies included 100 people and nine people.

**Table 1:** Characteristics and details of interventions of included studies.

**Reference**	**Sample size**	**Age**		**Duration (day)**		**Criteria for grading**	**Intervention**		**Intensity of treatment**	**Duration of treatment**	**Outcomes**
		**Experimental**	**Control**	**Experimental**	**Control**		**Experimental**	**Control**			
Aruin et al. (30)	18	Not described	Not described	Not described	Not described	Not described	CIMT + Conventional physiotherapy	Conventional physiotherapy	1 h/day	6 W	FMA, BBS, 10MWT, Weight Bearing
Qing and Yan (47)	120	61.38 ± 7.24	61.76 ± 7.83	4.52 ± 1.27(M)	4.65 ± 1.19(M)	E	CIMT	Conventional physiotherapy	6 h/day, 5 days/week	8 W	FMA-L, MBI, WHOQOL
Candan et al. (31)	30	55.13 ± 14.7	57.67 ± 12.2	6.80 ± 2.70(M)	6.63 ± 3.18(M)	I	mCIMT	Conventional physiotherapy	2 h/day, 5 days/week	2 W	BBS, GS
Candan et al. (32)	30	55.13 ± 14.7	57.67 ± 12.2	6.80 ± 2.70(M)	6.63 ± 3.18(M)	I	mCIMT	Conventional physiotherapy	2 h/day, 5 days/week	2 W	SSQOL
Cong et al. (33)	60	Not described	Not described	Not described	Not described	Not described	CIMT	Conventional physiotherapy	4 h/day, 5 days/week	8 W	FMA-L, BBS, MBI
Dou et al. (34)	58	55.1 ± 10.8	56.5 ± 14.5	26.7 ± 11.1(d)	28.2 ± 9.7(d)	A	mCIMT	Conventional physiotherapy	4 h/day, 5 days/week	8 W	FMA-L, BBS, 10MWT
EMGS et al. (26)	38	52~63	47~66	1~7(M)	2~7(M)	Not described	CIMT	Conventional physiotherapy	30 min/day, a total of 9 days	2 W	BBS, TUGT
Fang (35)	85	36 ± 4	37 ± 6	1 19(Y)	1 16(Y)	Not described	CIMT	Conventional physiotherapy	4 h/day, 5 days/week	2 M	10MWT, MBI
Guiyun et al. (36)	60	63.1 ± 13.18	61.9 ± 11.7	12.0 ± 8.5(d)	13.4 ± 7.6(d)	Not described	mCIMT	Conventional physiotherapy	6 h/day	8 W	BBS, TUGT, 10MWT
Junjie et al. (38)	122	56.49 ± 5.23	56.30 ± 4.98	22.29 ± 1.38(d)	22.33 ± 4.28(d)	A	CIMT + Western medicine	Western medicine	3 h/day	3 M	FMA-L, FAC, NIHSS
Jin et al. (37)	22	59.18 ± 7.34	58 ± 6.97	3.90 ± 0.83(M)	3.72 ± 0.78(M)	A	mCIMT + Conventional physiotherapy	Conventional physiotherapy	2 h/day,5 days/week	4 W	FMA-L, BBS, TUGT, 10MWT
Jung et al. (39)	21	56.4 ± 11.1	56.3 ± 17.1	6.2 ± 2.5(M)	7.0 ± 2.5(M)	J	CIMT	Conventional physiotherapy	30 min/d, 5 days/week	4 W	GS
Li et al. (40)	200	59.4 ± 9.7	61.3 ± 8.9	16.4 ± 9.3(d)	18.1 ± 9.0(d)	A	CIMT + Conventional physiotherapy	Conventional physiotherapy	40 min /once, 4 times/day, 6 days/week	6 W	FMA-L, BBS, MBI, FAC
Li (41)	60	62.34 ± 2.13	61.92 ± 2.09	Not described	Not described	Not described	CIMT	Conventional physiotherapy	4 h/day, 5 days/week	2 M	10MWT,MBI
Liang et al. (42)	96	56.4 ± 9.6	54.1 ± 10.2	20.3 ± 9.2(d)	21.2 ± 10.2(d)	A	Mcimt	Conventional physiotherapy	4 h/day	8 W	BBS, FMA-L, FAC, FIM
Liu et al. (43)	82	62.07 ± 4.15	62.05 ± 4.27	Not described	Not described	C	CIMT + Conventional physiotherapy	Conventional physiotherapy	1 h/day, 5 days/week	1 M	MBI, FMA-L, BBS, TUGT
Luo (44)	44	57.52 ± 11.31	57.67 ± 12.9	4.70 ± 1.02(W)	4.24 ± 1.00(W)	A	CIMT + Conventional physiotherapy	Conventional physiotherapy	6 h/day, 5 days/week	8 W	FMA-L, MBI, BBS, 6MWT
Mei (45)	28	Not described	Not described	Not described	Not described	Not described	CIMT	Conventional physiotherapy	4 h/day, 5 days/week	7 W	10MWT, BBS, FMA-L, MBI
Nie (46)	80	60.5 ± 1.3	60.2 ± 1.2	Not described	Not described	Not described	CIMT	Conventional physiotherapy	4 h/day, 5 days/week	4 W	10MWT, BBS, TUGT
Wang (48)	45	62.4 ± 12.5	65.2 ± 13.8	18.0 ± 7.7(d)	22.4 ± 4.7(d)	Not described	mCIMT	Conventional physiotherapy	6 h/day, 5 days/week	8 W	BBS, FMA-L
Wang (49)	94	53.8 ± 9.7	54.5 ± 9.4	2.9 ± 1.6(M)	2.7 ± 1.7(M)	B	CIMT	Conventional physiotherapy	2 h/day	4 W	FAC
Wang (52)	40	59.05 ± 6.01	59.10 ± 7.99	5.8 ± 2.04(M)	5.9 ± 1.77(M)	F	CIMT + Conventional physiotherapy	Conventional physiotherapy	2 h/day,5 days/week	8 W	6MWT, TUGT, MBI
Wang (50)	30	63.1 ± 13.2	61.9 ± 11.7	12.0 ± 8.5(d)	13.4 ± 7.6(d)	A	CIMT	Conventional physiotherapy	4 h/day, 5 days/week	8 W	10MWT, BBS, FMA-L, MBI
Wang (51)	67	73.2 ± 5.2	76.4 ± 3.8	Not described	Not described	D	mCIMT	Conventional physiotherapy	4 h/day, 5 days/week	6 W	FMA-L, BBS, 10MWT, TUGT
Wu et al. (54)	36	63.94 ± 13.78	66.17 ± 13.99	Not described	Not described	A	CIMT + Conventional physiotherapy	Conventional physiotherapy	40 min/once,4 times/day,6 days/week	4 W	MBI, FAC, Brunnstrom stage of lower limb function
Xiaoru et al. (55)	64	55.1 ± 10.8	56.5 ± 14.5	Not described	Not described	A	mCIMT	Conventional physiotherapy	4 h/day, 5 days/week	8 W	FMA-L, BBS
Yin et al. (56)	30	61.3 ± 13.4	59.8 ± 12.6	150 ± 30(d)	146 ± 32(d)	A	CIMT	Conventional physiotherapy	4 h/day, 5 days/week	4 W	FMA, BBS, MBI
Yu et al. (57)	21	54.2 ± 11.1	56.8 ± 11.0	1.0 ± 0.5(Y)	1.2 ± 1.3(Y)	I	CIMT	Conventional physiotherapy	90 min/day, 5 days/week	2 W	GS, TUGT, SSQOL
Wei ming et al. (53)	60	60.17 ± 2.73	58.27 ± 6.75	4.90 ± 1.90(d)	4.13 ± 2.10(d)	A	mCIMT	Conventional physiotherapy	60 min/once, twice/day, 5 days/week	2 W	FMA-L, BBS, MBI
Zhang (58)	52	62.32 ± 3.65	61.98 ± 4.54	Not described	Not described	Not described	CIMT	Conventional physiotherapy	4 h/day, 5 days/week	6 W	10MWT, BBS, TUGT
Zhou (59)	104	49.1 ± 3.2	48.6 ± 3.4	Not described	Not described	Not described	CIMT + Conventional physiotherapy	Conventional physiotherapy	2 h/day, 5 days/week	4 W	BBS, FAC
Zhu (60)	40	59.05 ± 6.01	59.1 ± 7.99	79 ± 9.56(d)	78.45 ± 9.05(d)	G	mCIMT + Conventional physiotherapy	Conventional physiotherapy	2 h/day, 5 days/week	8 W	FMA-L, BBS, MBI, TUGT, 10MWT,6MWT
Zhu (62)	49	Not described	Not described	Not described	Not described	I	CIMT	Conventional physiotherapy	4 h/day, 5 days/week	6 W	10MWT, BBS, TUGT
Zhu (61)	22	59.18 ± 7.34	58 ± 6.97	3.90 ± 0.83(M)	3.72 ± 0.78(M)	G	mCIMT	Conventional physiotherapy	2 h/day, 5 days/week	4 W	GS

d, day; W, week; M, mounth; Y, year; FMA, Fugel-Meyer Assessment scale; FMA-L, Fugel-Meyer Assessment scale on Lower limbs; MBI, Modified Barthel Index scale; BBS, Berg Balance Scale; 10MWT: 10 Meter Walk Test; TUGT, Timed Up and Go Test; 6MWT: 6 min Walk Test; GS, Gait Speed; FAC, Functional Ambulation Category scale; FIM, Functional Independence Measure; WHOQOL, World Health Organization Quality of Life; SSQOL, Stroke Specific Quality of Life scale; NIHSS, National Institute of Health stroke scale.

A, Diagnostic criteria formulated by the National Cerebrovascular Disease Conference; B, Guidelines for Prevention and Treatment of Cerebrovascular Diseases in China; C, Diagnostic points of various cerebrovascular diseases; D, Guidelines for Secondary Prevention of Ischemic Stroke and Transient Ischemic Attack in China 2010; E, Diagnosis and therapy of stroke; F, Chinese Classification of Cerebrovascular Diseases 2015; G, Brunnstrom rated in II-III; I, Brunnstrom rated in III-V; J, functional ambulation classification in II-III.

In terms of intervention, 24 studies adopted CIMT, nine studies adopted CIMT in combination with conventional physiotherapy (such as muscle strengthening, facilitation, activity training, balance, gait training, or neurodevelopmental treatment), and one study adopted CIMT in combination with western medicine (including improving cerebral blood circulation, nerve nutrition, and blood pressure regulation). Table 2 lists the CIMT protocol details of the included studies.

**Table 2:** Details of the CIMT scheme.

**References**	**CIMT scheme of studies**
Aruin et al. (30)	Forced shift of body weight toward a person's affected side by means of the 0.6-cm full-shoe insoles insert that establishes a lift of the nonaffected lower extremity. Muscle-strengthening activities included progressive resistance exercises with Thera-Band resistive bands and the NuStep exercise machine. Weight-bearing exercises involved a bathroom scale, sit-to-stand and stand-to-sit practice with emphasis on equal weight bearing on both sides. Balance exercises included weight shifts on the affected side and pregait activities such as stepping forward, stepping sideways, and stepping on a stool.
Qing and Yan (47)	The training included: balance exercises, treadmill training, sit-to-stand transfers, one-leg weight bearing, climbing stairs.
Candan et al. (31)	mCIMT included: the intensive practice of the functional activities, limited use of the nonparetic lower limb (by whole leg orthosis and 1cm shoe raise) and transferring the gains from the training session to the patient's real environment with “transfer package”.
Candan et al. (32)	mCIMT included: the intensive practice of the functional activities, limited use of the nonparetic lower limb (by whole leg orthosis and 1cm shoe raise) and transferring the gains from the training session to the patient's real environment with “transfer package”.
Cong et al. (33)	The nonaffected lower extremity wore the modified knee splint, and the daily wearing time was not less than 90% of the waking time. The training included: sit-to-stand transfers, standing training, gait training, treadmill training, and climbing stairs.
Dou et al. (34)	Limb was fixed to limit the use of the nonparetic lower limb. The training included: one-leg weight bearing, walk over obstacles, sit-to-stand transfers, quadriceps closed chain training, and balance exercises.
EMGS et al. (26)	The experimental group performed treadmill training, but using a mass attached around the non-paretic ankle, with load equivalent to 5% of the individual body weight.
Fang (35)	The training included: treadmill training, rehabilitation of bicycle endurance and quadriceps resistance training, sit-to-stand transfers, one-leg weight bearing, balance exercises, climbing stairs and indoor walking.
Guiyun et al. (36)	The training included: treadmill training, sit-to-stand transfers, one-leg weight bearing, balance exercises, climbing stairs and indoor walking.
Junjie et al. (38)	The training included: sit-to-stand transfers, gait training (Swing back and forth, crotch extension, stepping, knee flexion exercises and bearing weight were performed on the affected leg, the healthy leg moving slowly forward)
Jin (37)	The training included: sit-to-stand transfers, indoor walking, one leg was loaded on the affected side, balance exercises and climbing stairs.
Jung et al. (39)	Gait training using a cane with an augmented pressure sensor to enhance weight bearing over the affected lower limb. The cane can provide auditory feedback.
Li et al. (40)	The training included: sit-to-stand transfers, one-leg weight bearing, balance exercises and climbing stairs.
Li (41)	The training included: treadmill training, rehabilitation of bicycle endurance, sit-to-stand transfers, one-leg weight bearing, balance exercises, climbing stairs and outdoor walking.
Liang et al. (42)	The training included: treadmill training, sit-to-stand transfers, one-leg weight bearing, balance exercises, climbing stairs and outdoor walking.
Liu et al. (43)	A splint was used to restrict the movement of nonparetic lower limb. The time was at least 90% of the waking time. The training included: treadmill training, sit-to-stand transfers and climbing stairs.
Luo (44)	The nonparetic lower limb wore the modified knee splints for no less than 90% of the waking time every day. The training included: treadmill training, sit-to-stand transfers, gait training and climbing stairs.
Mei (45)	The training included: treadmill training, sit-to-stand transfers, balance exercises, one-leg weight bearing, outdoor walking and climbing stairs.
Nie (46)	The training included: treadmill training, sit-to-stand transfers, gait training, climbing stairs, balance exercises and one-leg weight bearing.
Wang (48)	The training included: treadmill training, sit-to-stand transfers, climbing stairs, balance exercises, outdoor walking and one-leg weight bearing.
Wang (49)	The training included: sit-to-stand transfers, balance exercises, gait training, one-leg weight bearing and climbing stairs.
Wang et al. (52)	The training included: sit-to-stand transfers, balance exercises, gait training, one-leg weight bearing and climbing stairs.
Wang et al. (50)	The training included: treadmill training, sit-to-stand transfers, balance exercises, quadriceps resistance training, one-leg weight bearing, outdoor walking and climbing stairs.
Wang et al. (51)	The training included: treadmill training, sit-to-stand transfers, balance exercises, quadriceps resistance training, one-leg weight bearing, and rehabilitation with bicycle.
Wu et al. (54)	The training included: weight support plate training, gait training, center of gravity transfer training.
Xiaoru et al. (55)	Limb was fixed to limit the use of the nonparetic lower limb. The training included: one-leg weight bearing, walk over obstacles, sit-to-stand transfers, quadriceps closed chain training, and balance exercises.
Yin et al. (56)	The nonparetic lower limb wore the modified knee splints for no less than 90% of the waking time every day. The training included: treadmill training, sit-to-stand transfers, gait training and climbing stairs.
Yu et al. (57 )	Participants wore a custom-fitted wedged insole under the unaffected side to force the use of the affected limb. The training program included: sit-to-stand, stepping over blocks in different directions, walking on an inclined treadmill, climbing stairs, and walking over various surfaces with obstacles. The physical therapist encouraged maximal usage of the affected limb while performing those tasks.
Wei ming et al. (53)	Verbal reminders were used to restrict the movement of the less-affected limb, but no fixation device was worn. The patient was asked to minimize the use and assistance of the less-affected limb during the waking time. The training included: sit-to-stand transfers, one-leg weight bearing, gait training and balance exercises.
Zhang (58)	The training included: sit-to-stand transfers, treadmill training, outdoor walking, climbing stairs and balance exercises.
Zhou (59)	The training included: sit-to-stand transfers, balance exercises, weight training for both lower limbs, hip alignment training, quadriceps closed chain training and gait training.
Zhu (60)	The training included: sit-to-stand transfers, gait training, treadmill training, climbing stairs and balance exercises.
Zhu (62)	The training included: sit-to-stand transfers, treadmill training, outdoor walking, climbing stairs, balance exercises and one-leg weight bearing.
Zhu (61)	mCIMT gait training for about 2 h included: sit-to-stand transfers, indoor walking, climbing up and down stairs training, balance training, one leg weight training on paretic and non-paretic leg and muscle strength training.

Regarding outcome assessment, 18 studies assessed lower extremity motor function using the FMA scale (of which 16 assessed FMA-L). Additionally, 23 studies assessed the balance function through the BBS scale. Concerning walking speed evaluation, 13 studies measured 10MWT score, and 4 measured GS. Regarding mobility assessment, 11 studies used TUGT, 6 used FAC, 3 used 6MWT, and 1 used Brunnstrom Stage of lower limb function. To assess activities of daily living, 14 studies used MBI and 1 used FIM. As for quality of life assessment, two studies used SSQOL and one used WHOQOL. One study used the NIHSS to measure the severity of stroke damage. Only one study evaluated the weight bearing of affected limbs.

### Methodological quality

The risk of bias in the 34 studies is shown in Supplementary Figures S2, S3. All 34 studies randomly divided the participants into different groups. Five studies (26, 31, 32, 39, 57) detailed assignment protocol concealment and four studies (31, 32, 44, 60) reported non-blindness by trial personnel (the first author performed the CIMT treatment). In addition, two studies (44, 53) were performed without blindness to the outcome assessment, and 16 (26, 31–34, 36, 37, 39, 40, 48, 50, 51, 55, 57, 61, 62) reported outcome assessments completed by therapists who did not participate in the experiment. Finally, 11 studies (34, 36, 40, 42, 45, 48, 53, 54, 56, 60, 62) did not clearly record whether the baseline was balanced and were, therefore, rated “unclear”. We assessed the quality of the included studies using the Cochrane Bias Risk Assessment tool and found methodological defects. Only four studies (26, 39, 57, 61) were rated “high quality”.

### Effect of CIMT: Clinical evaluation

#### CIMT vs. conventional physiotherapy

##### Effect on lower extremity motor function

**Figure 2:**
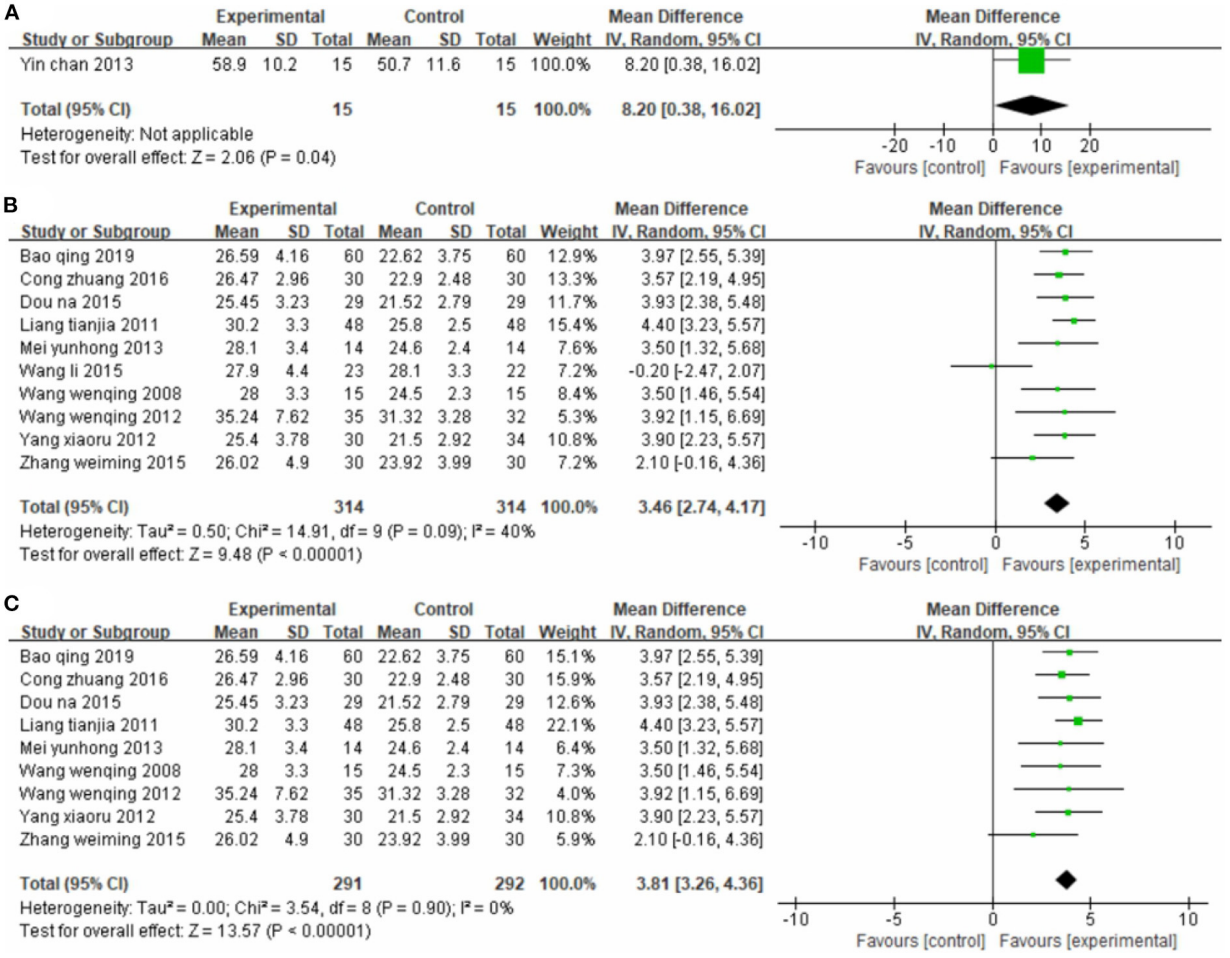
Forest plot. CIMT vs. conventional physiotherapy on the Fugl-Meyer Assessment total score **(A)** and Fugl-Meyer Assessment lower limb sub-scale **(B,C)**.

One study (56) used FMA as the outcome indicator (Figure 2A). It included 30 cases. Its MD value was 8.20 (95% CI 0.38–16.02, P = 0.04).

Ten studies (33, 34, 42, 45, 47, 48, 50, 51, 53, 55), totaling 628 participants, reported the FMA-L (Figure 2B). Our pooled data analysis showed that the CIMT group had a higher FMA-L evaluation score than the conventional physiotherapy group, with an MD value of 3.46 (95% CI 2.74–4.17, *P* < 0.01, I^2^ = 40%). The sensitivity analysis showed that one study (48) was the main source of heterogeneity (Figure 2C). Removing this study notably reduced the interstudy heterogeneity (*P* = 0.90, I^2^ = 0%) but yielded similar analysis results (MD = 3.81, 95% CI 3.26–4.36, *P* < 0.01).

##### Effect on balance function

**Figure 3:**
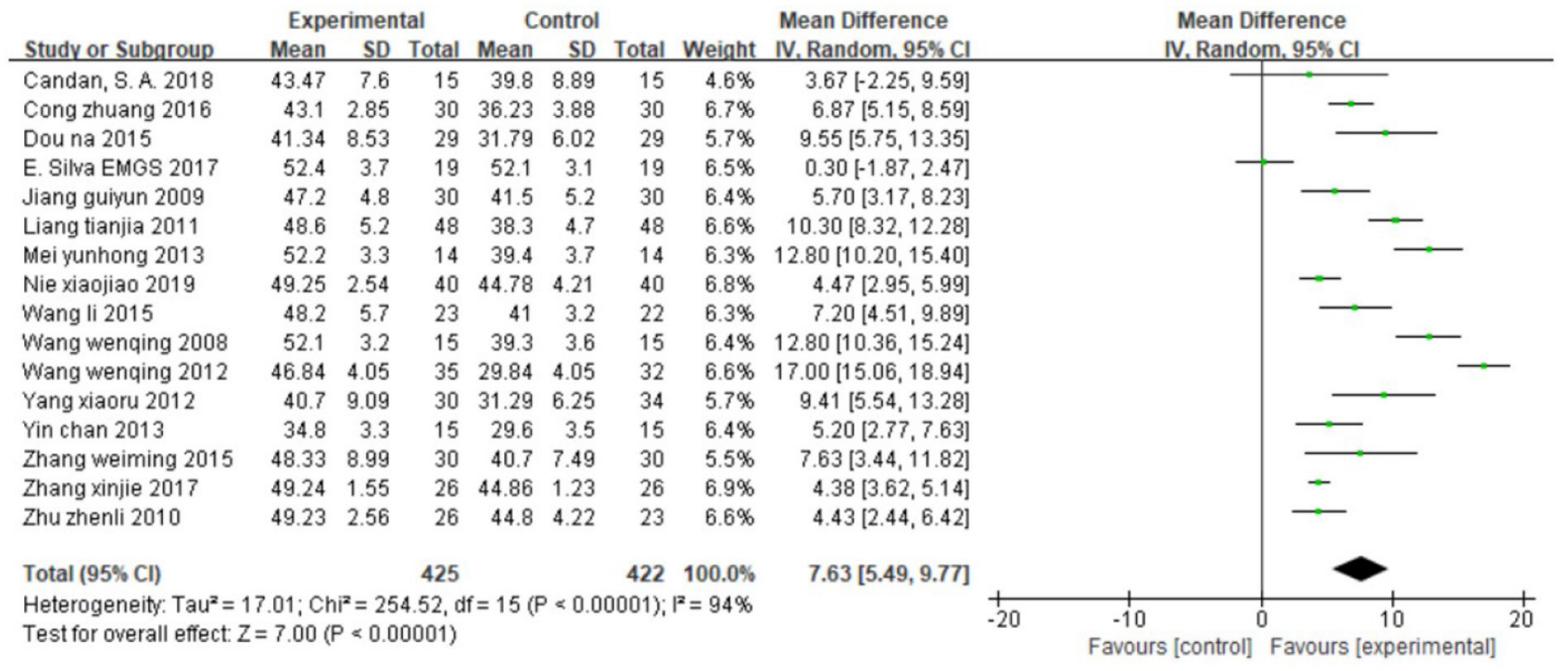
Forest plot. CIMT vs. conventional physiotherapy on the Berg balance scale.

Sixteen studies (26, 31, 33, 34, 36, 42, 45, 46, 48, 50, 51, 53, 55, 56, 58, 62), totaling 847 cases, used BBS as the outcome indicator (Figure 3). The MD value was 7.63 (95% CI 5.49–9.77, *P* < 0.01, I^2^ = 94%), indicating that CIMT was superior to conventional physiotherapy in improving balance function. However, these studies were strongly heterogeneous. Unfortunately, neither the sensitivity analysis nor the meta-regression analysis of publication year and sample size reveals any find obvious sources of heterogeneity. We suspect publication bias because the funnel plot was asymmetric, and eight studies were outside it (Supplementary Figure S4). We also confirmed publication bias in this analysis using Egger's test (*P* = 0.02, Supplementary Figure S5).

##### Effect on walking speed

**Figure 4:**
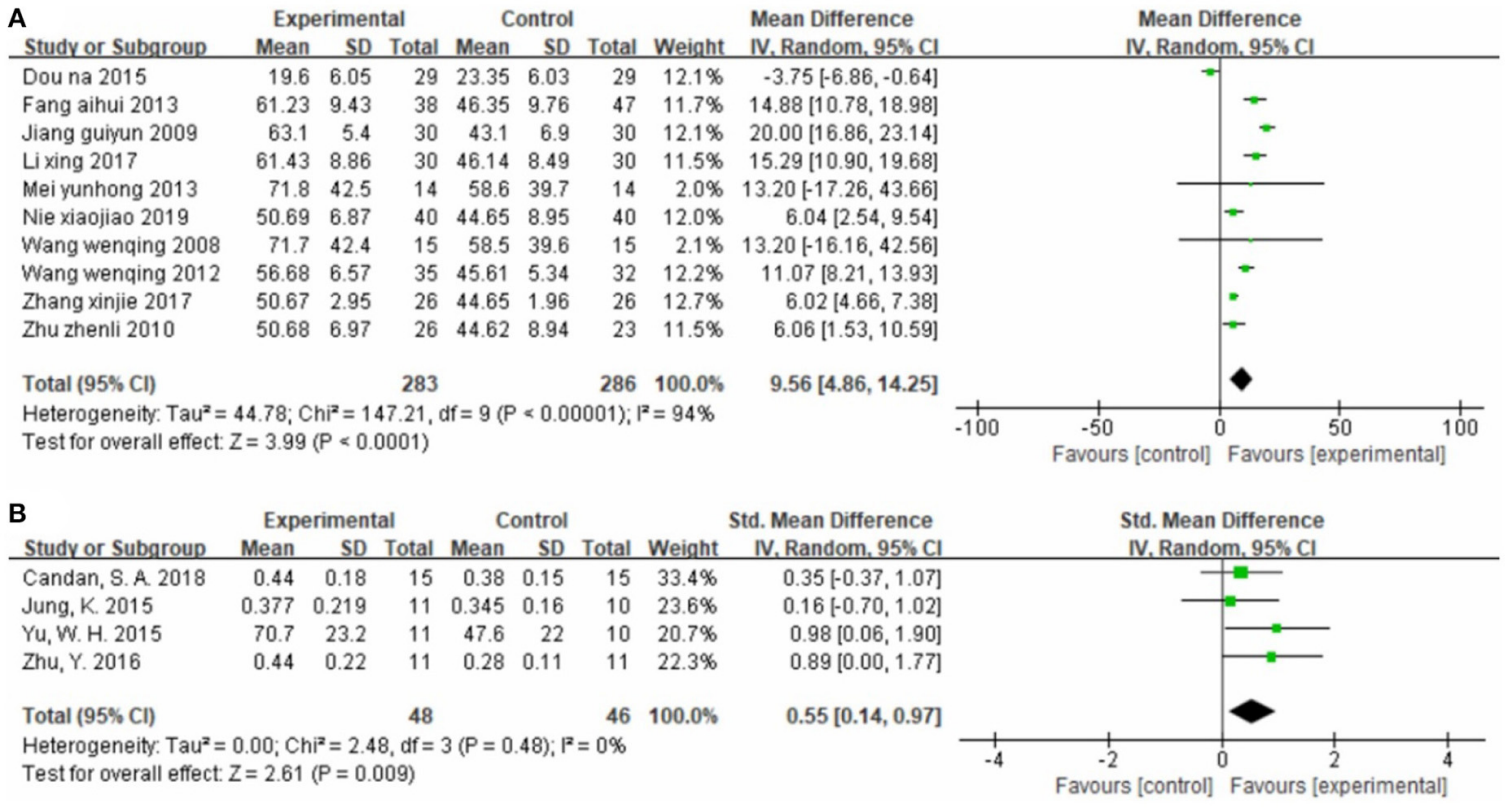
CIMT vs. conventional physiotherapy on the 10-meter walk test **(A)** and gait speed **(B)**.

Ten studies (34–36, 41, 45, 46, 50, 51, 58, 62), totaling 569 cases, assessed motor function using 10MWT (Figure 4A) (MD = 9.56, 95% CI 4.86–14.25, *P* < 0.01, I^2^ = 94%). Neither the sensitivity analysis nor the meta-regression analysis of publication year and sample size revealed any obvious sources of heterogeneity. As shown in Supplementary Figure S6, the funnel plot for this analysis is symmetric. Egger's test confirmed the symmetry of the funnel plot, and we found no evident publication bias in this analysis (*P* = 0.351) (Supplementary Figure S7).

Four studies (31, 39, 57, 61), totaling 94 cases, measured GS as the outcome indicator (Figure 4B). The standard MD was 0.55 (95% CI 0.14–0.97, *P* < 0.01, I^2^ = 0%), indicating that CIMT is superior to conventional physiotherapy for improving walking speed.

##### Effect on mobility

**Figure 5:**
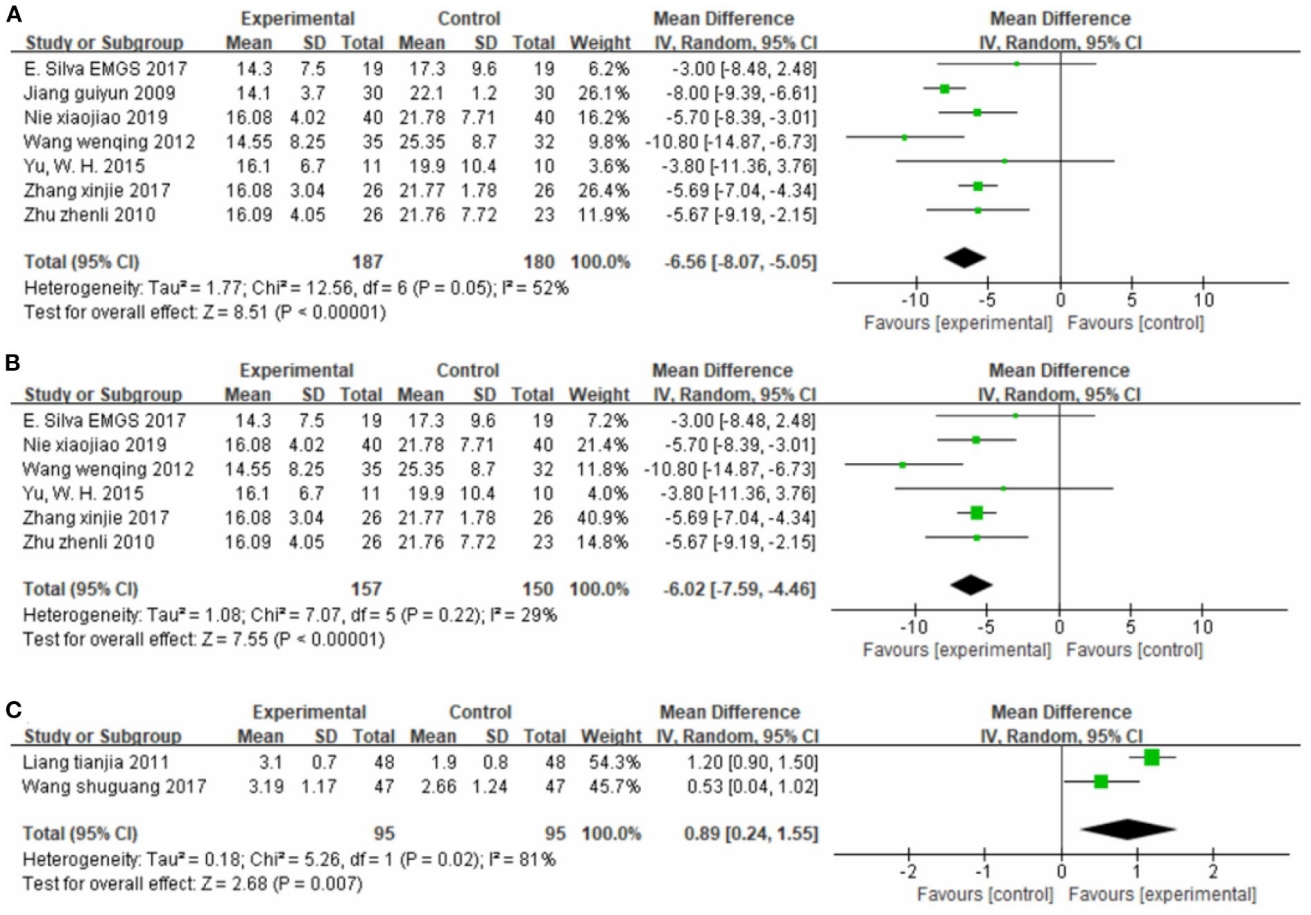
Forest plot. CIMT vs. conventional physiotherapy on the timed up and go test **(A,B)** and functional ambulation category scale **(C)**.

Seven studies (26, 36, 46, 51, 57, 58, 62), totaling 367 cases, measured the TUGT score as the outcome indicator (Figure 5A). Our pooled data analysis showed that patients in the experimental group spent less time on the TUGT, with an MD value of −6.56 (95% CI −8.07 to −5.05, *P* < 0.01, I^2^ = 52%). The sensitivity analysis revealed that one study (36) was the main source of heterogeneity (Figure 5B). Removing it markedly reduced heterogeneity (P = 0.22, I^2^ = 29%), and yielded an MD value of −6.02 (95% CI −7.59 to −4.46, *P* < 0.01).

Two studies (42, 49), totaling 190 cases, used FAC as the outcome indicator (Figure 5C). The MD value was 0.89 (95% CI 0.24–1.55, *P* < 0.01, I^2^ = 81%). The above results indicate that CIMT is better than conventional physiotherapy for improving patients' mobility, although there was some heterogeneity.

##### Effect on activities of daily living

**Figure 6:**
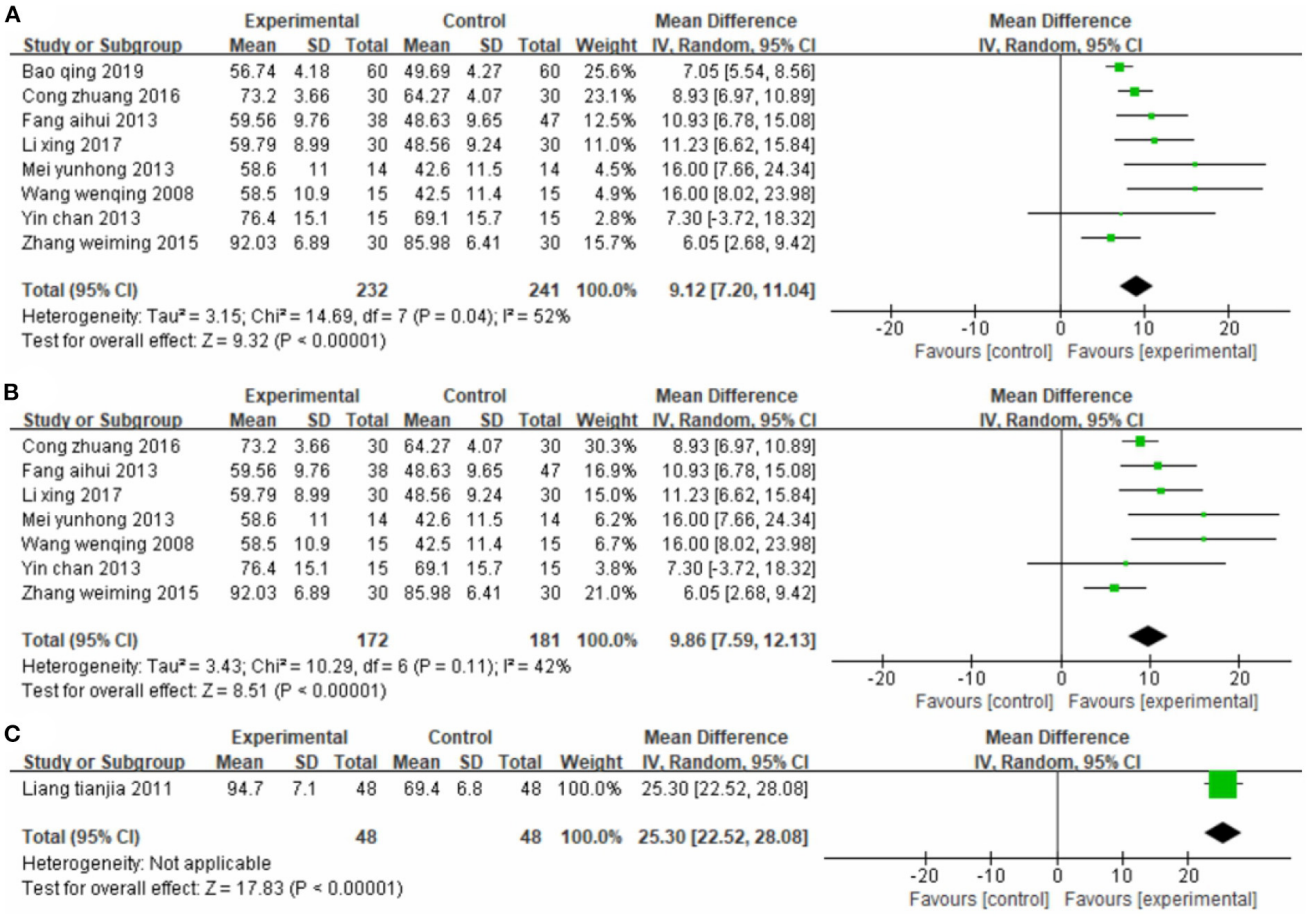
Forest plot. CIMT vs. conventional physiotherapy on the modified Barthel index **(A,B)** and functional independence measure **(C)**.

Eight studies (33, 35, 41, 45, 47, 50, 53, 56), totaling 473 cases, used MBI as the outcome indicator (Figure 6A). The MD value was 9.12 (95% CI 7.20–11.04, *P* < 0.01, I^2^ = 52%). The meta-regression analysis of publication year and sample size did not reveal obvious sources of heterogeneity. However, the sensitivity analysis identified one study (47) as the main source of heterogeneity (Figure 6B). Removing it notably reduced heterogeneity (P = 0.11, I^2^ = 42%) and yielded an MD value of 9.86 (95% CI 7.59–12.13, *P* < 0.01).

One study (42), totaling 96 cases, used FIM as the outcome indicator (Figure 6C). The MD value was 25.30 (95% CI 22.52–28.08, *P* < 0.01). The above results indicate that patients in the CIMT group had higher scores than those in the conventional physiotherapy group.

##### Effect on quality of life

**Figure 7:**
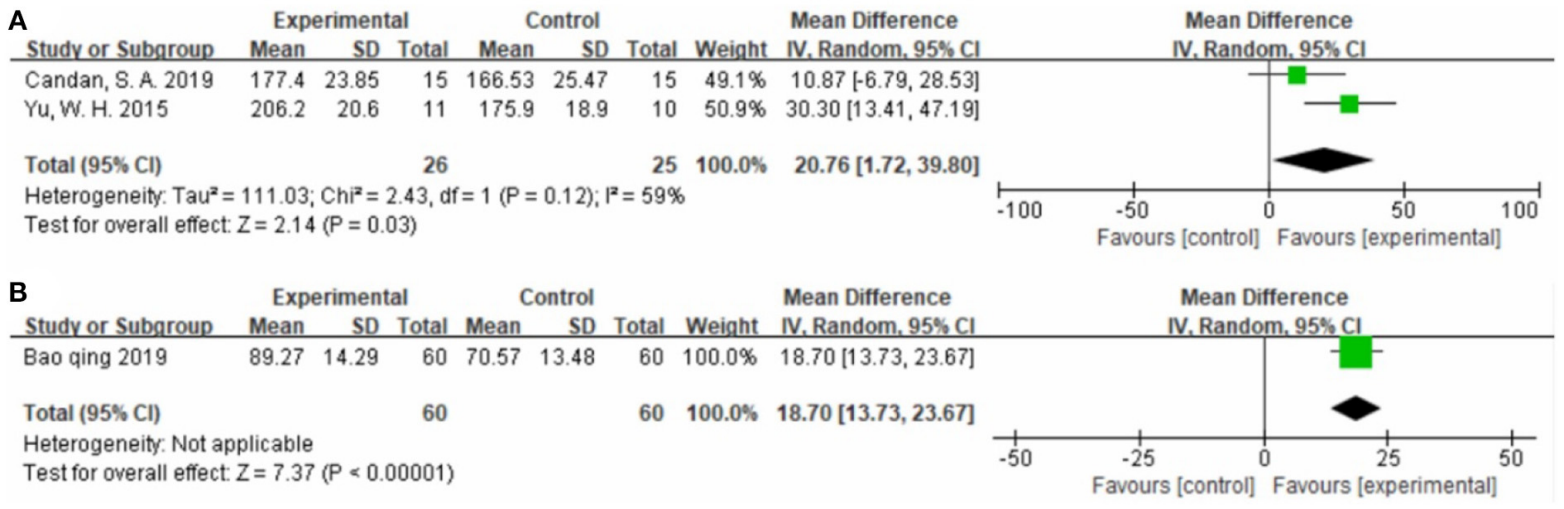
Forest plot. CIMT vs. conventional physiotherapy on the stroke-specific quality of life questionnaire **(A)** and World Health Organization quality of life assessment **(B)**.

Two studies (32, 57), totaling 51 cases, used SSQOL as the outcome indicator (Figure 7A). The MD value was 20.76 (95% CI 1.72–39.80, P = 0.03, I^2^ = 59%). One study (47), totaling 120 cases, used WHOQOL as the outcome indicator (Figure 7B). The MD value was 18.70 (95% CI 13.73–23.67, *P* < 0.01). The above results indicate that patients treated with CIMT had higher Quality of Life scores than patients treated with conventional physiotherapy.

#### CIMT plus conventional physiotherapy vs. conventional physiotherapy

##### Effect on lower limb motor function

**Figure 8:**
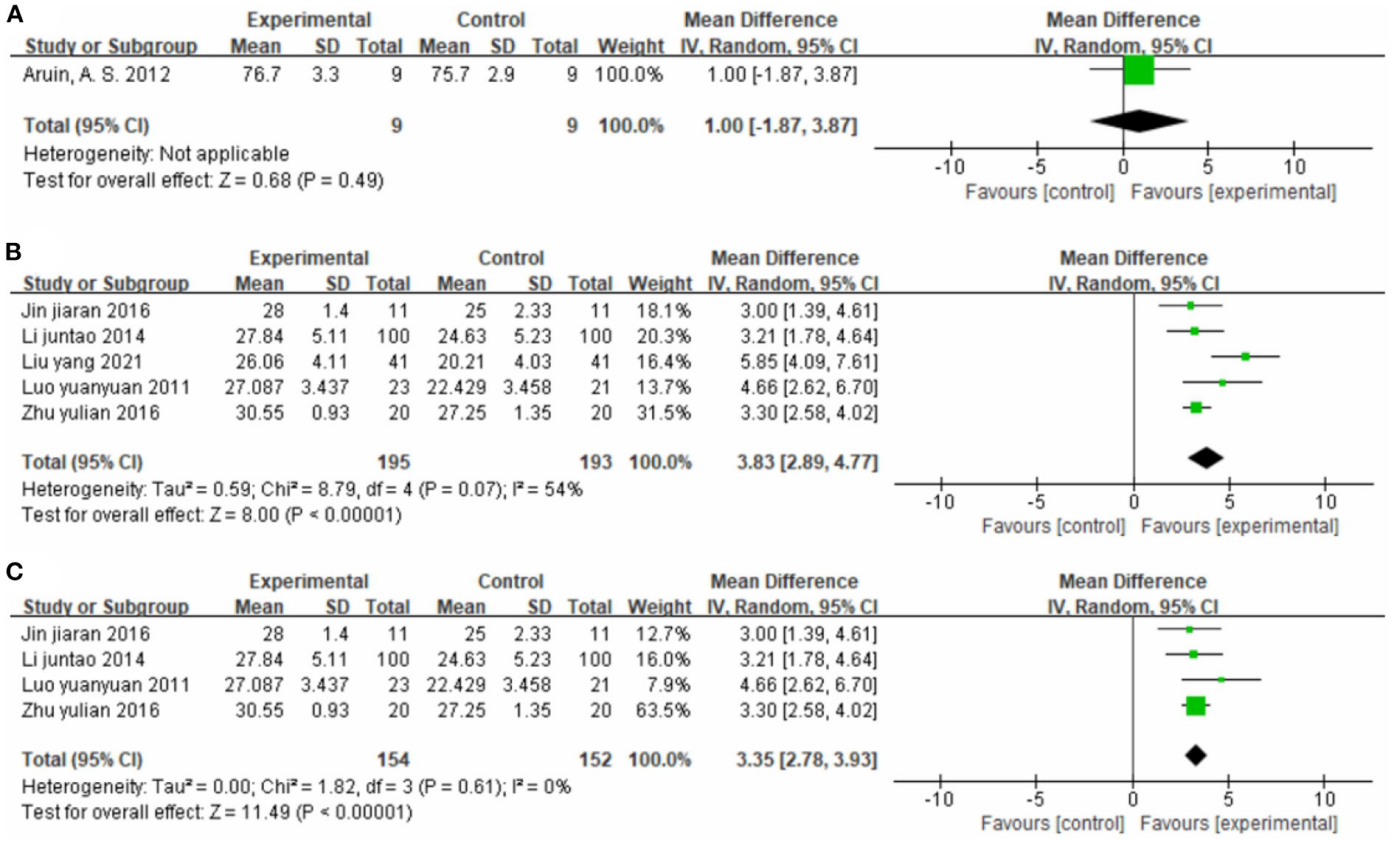
Forest plot. CIMT plus conventional physiotherapy vs. conventional physiotherapy alone on the Fugl-Meyer Assessment total score **(A)** and Fugl-Meyer Assessment lower limb sub-scale **(B,C)**.

One study (30), totaling 18 cases, used FMA as the outcome indicator (Figure 8A). The MD value was 1.00 (95% CI −1.87 to 3.87, P = 0.49).

Five studies (37, 40, 43, 44, 60), totaling 388 participants, used the FMA-L as the outcome indicator (Figure 8B). The pooled analysis showed that CIMT combined with conventional physiotherapy was better than conventional physiotherapy alone for improving the motor function of the patients' lower limb (MD = 3.83, 95% CI 2.89–4.77, *P* < 0.01, I^2^ = 54%). The sensitivity analysis identified one study (43) as the main source of heterogeneity (Figure 8C). Removing it markedly reduced the heterogeneity (*P* = 0.61, I^2^ = 0%), and yielded similar results (MD = 3.35, 95% CI 2.78–3.93, *P* < 0.01).

##### Effect on balance function

**Figure 9:**
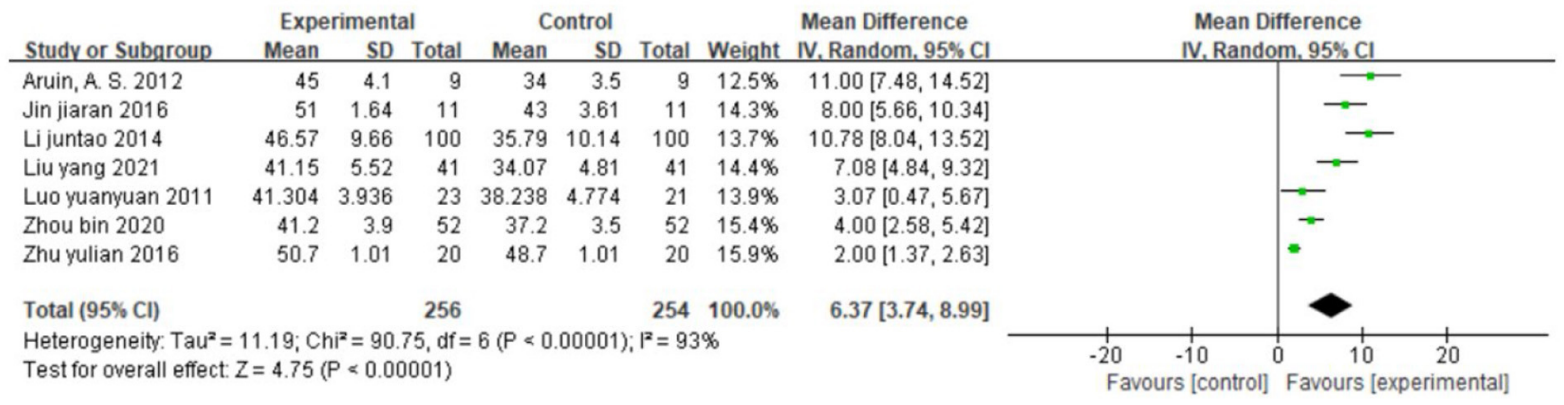
Forest plot. CIMT plus conventional physiotherapy vs. conventional physiotherapy alone on the Berg balance scale.

Seven studies (30, 37, 40, 43, 44, 59, 60), totaling 510 cases, used BBS as the outcome indicator (Figure 9). The MD value was 6.37 (95% CI 3.74–8.99, *P* < 0.01, I^2^ = 93%), indicating that CIMT combined with conventional physiotherapy was superior to Conventional physiotherapy alone for improving the balance function. However, the studies were strongly heterogeneous and the meta-regression analysis did not reveal publication year and sample size as obvious sources of heterogeneity.

##### Effect on walking speed

**Figure 10:**
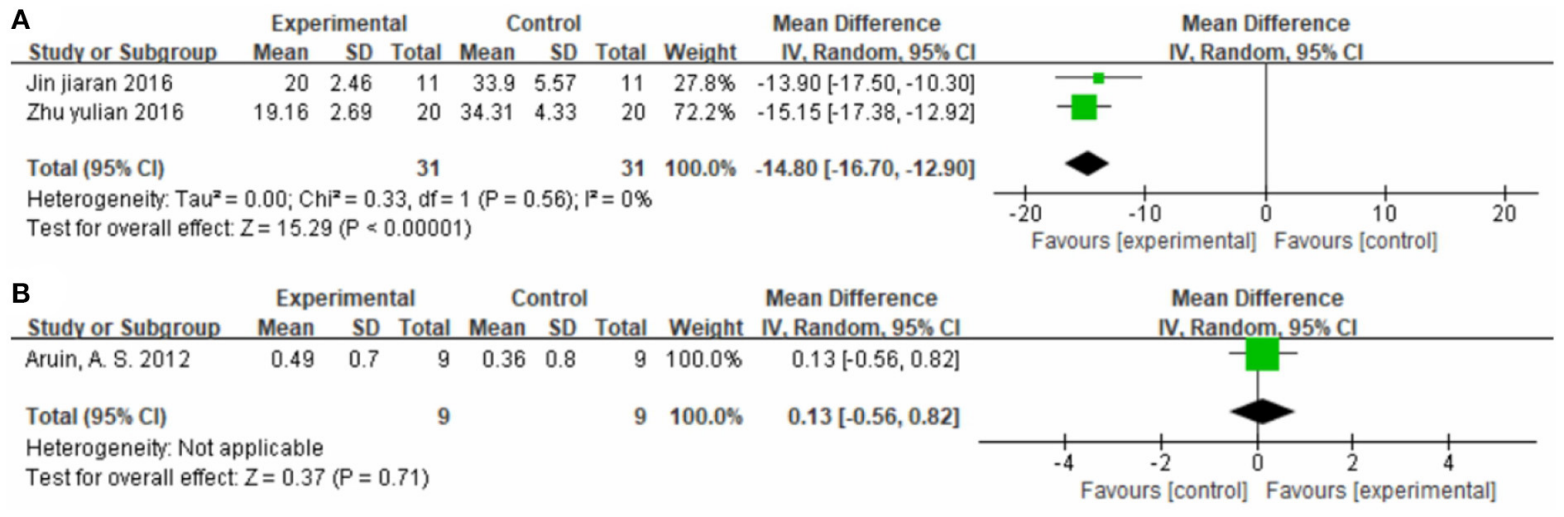
Forest plot. CIMT plus conventional physiotherapy vs. conventional physiotherapy alone on the 10-meter walk test [**(A)** s; **(B)** m/s].

Two studies (37, 60), totaling 62 cases, measured 10MWT score (s) as the outcome indicator (Figure 10A). The patients in the experimental group spent less time on the 10-MWT than patients in the control group (MD = −14.80, 95% CI −16.70 to −12.90, *P* < 0.01, I^2^ = 0%). One study (30), totaling 18 cases, measured 10MWT score (m/s) as the outcome indicator included (Figure 10B). The MD value was 0.13 (95% CI −0.56 to 0.82, P = 0.71). The above results indicate that CIMT combined with conventional physiotherapy was better than conventional physiotherapy alone for improving walking speed.

##### Effect on mobility

**Figure 11:**
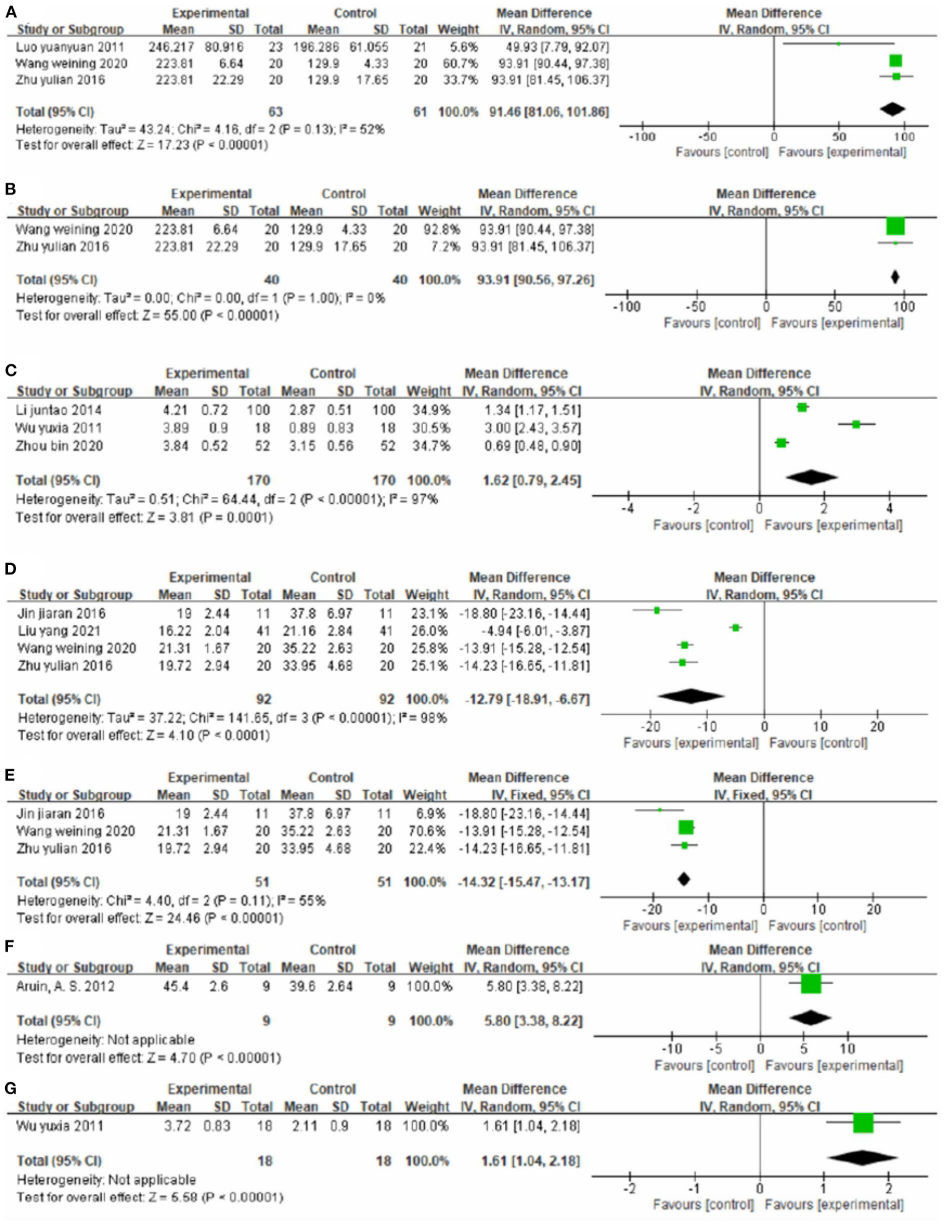
Forest plot. CIMT plus conventional physiotherapy vs. conventional physiotherapy alone on the 6-min walk test **(A,B)**, functional ambulation category scale **(C)**, timed up and go test **(D,E)**, weight-bearing **(F)** and Brunnstrom stage of lower limb function **(G)**.

Three studies (44, 52, 60), totaling 123 cases, used 6MWT score as the outcome indicator in Figure 11A. The experimental group walked longer distances within 6 min than the control group, with an MD value of 91.46 (95% CI 81.06–101.86, *P* < 0.01, I^2^ = 52%). The sensitivity analysis revealed that one study (44) was the main source of heterogeneity (Figure 11B). Removing it notably reduced the heterogeneity (P = 1.00, I^2^ = 0%) and yielded an MD value of 93.91 (95% CI 90.56–97.26, *P* < 0.01).

Three studies (40, 54, 59), totaling 340 cases, used FAC as the outcome indicator (Figure 11C). The MD value was 1.62 (95% CI 0.79–2.45, *P* < 0.01, I^2^ = 97%). The meta-regression analysis of publication year and sample size did not identify obvious sources of heterogeneity.

Four studies (37, 43, 52, 60), totaling 184 cases, measured TUGT score as the outcome indicator (Figure 11D). The patients in the experimental group had lower scores than control patients (MD = −12.79, 95% CI −18.91 to −6.67, *P* < 0.01, I^2^ = 98%). The sensitivity analysis revealed that one study (43) was the main source of heterogeneity (Figure 11E). Removing it markedly reduced heterogeneity (*P* = 0.11, I^2^ = 55%) and yielded an MD value of −14.32 (95% CI −15.47 to −13.17, *P* < 0.01).

One study (30), totaling 18 cases, measured Weight Bearing as the outcome indicator (Figure 11F). The lower limb weight was more balanced in the experimental group than in the control group (MD = 5.80, 95% CI 3.38–8.22, *P* < 0.01).

One study (54), totaling 36 cases, used Brunnstrom stage of lower limb function as the outcome indicator (Figure 11G). The MD value was 1.61 (95% CI 1.04–2.08, *P* < 0.01). The above results indicate that CIMT combined with conventional physiotherapy is better than conventional physiotherapy alone for improving the mobility of patients.

##### Effect on activities of daily living

**Figure 12:**
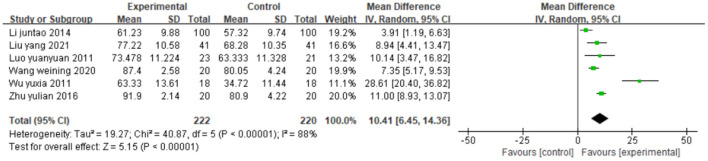
Forest plot. CIMT plus conventional physiotherapy vs. conventional physiotherapy alone on the modified Barthel index.

Six studies (40, 43, 44, 52, 54, 60), totaling 442 cases, used MBI as the outcome indicator (Figure 12). The patients in the experimental group had higher scores than control patients (MD = 10.41, 95% CI 6.45–14.36, *P* < 0.01, I^2^ = 88%). The meta-regression analysis of publication year and sample size did not find obvious sources of heterogeneity.

#### CIMT plus western medicine therapy vs. western medicine therapy

**Figure 13:**
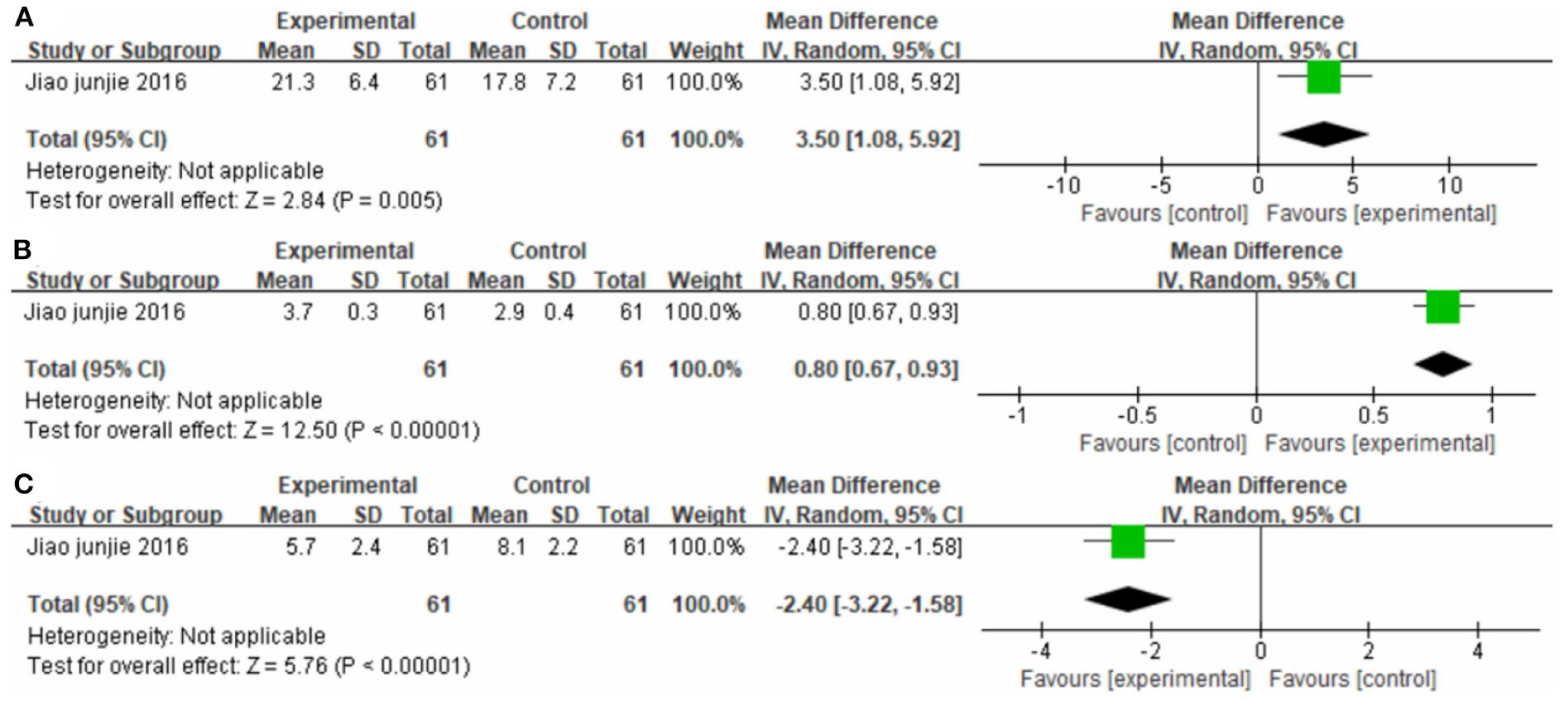
Forest plot. CIMT plus western medicine therapy vs. western medicine therapy alone on the Fugl-Meyer Assessment lower limb sub-scale **(A)**, functional ambulation category scale **(B)** and National Institute of Health stroke scale **(C)**.

One study (38), totaling containing 61 cases compared the efficacy of CIMT plus western medicine therapy with western medicine therapy alone (Figure 13). The MD values for FMA-L, FAC, and NIHSS were 3.50 (95% CI 1.08–5.92, *P* < 0.01); 0.80 (95% CI 0.67–0.93, *P* < 0.01); and −2.40 (95% CI −3.22 to −1.58, *P* < 0.01), respectively.

### Lower extremity CIMT with device vs. lower extremity CIMT without device

**Figure 14:**
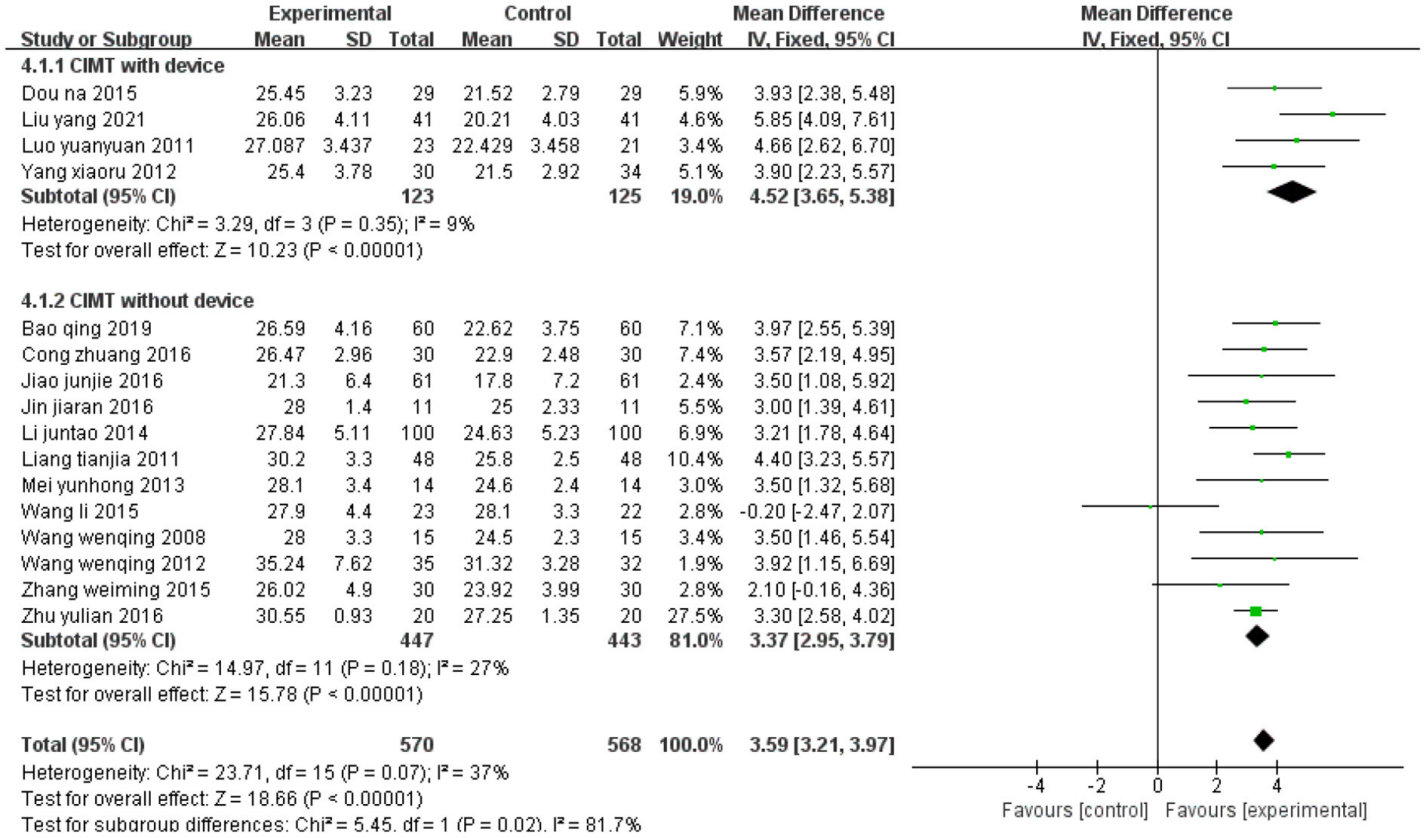
Forest plot. CIMT with device vs. CIMT without device on the Fugl-Meyer Assessment lower limb sub-scale.

Figure 14 shows the synthetic results on FMA-L from 16 studies. Four studies (34, 43, 44, 55) evaluated lower extremity CIMT with device, and 12 studies (33, 37, 38, 40, 42, 45, 47, 48, 50, 51, 53, 60) evaluated lower extremity CIMT without device. The subgroup analysis suggests that lower extremity CIMT with device yields higher FMA-L scores than lower extremity CIMT without device (With device: MD = 4.52, 95% CI 3.65 to 5.38, *P* < 0.01; without device: MD = 3.37, 95% CI 2.95 to 3.79, *P* < 0.01).

## Discussion

Although the effect of CIMT on post-stroke upper limb motor dysfunction has been widely studied, its effect on lower limbs remains incompletely understood. Fuzaro and co-workers (23) demonstrated that Forced Use Therapy and CIMT improve balance and gait, while lower extremity CIMT improves motor behavior patterns and scores on the functional scale. Silva and co-workers (26) observed that 2 weeks of treadmill gait training (with load to restrain the healthy lower limb), combined with home exercise, improved postural balance and functional flexibility in stroke patients. However, they concluded that the increased load in CIMT did not provide additional benefits for training. Auwal et al. (25) recently conducted a systematic evaluation and meta-analysis of CIMT in lower limbs, but their conclusion slightly differs from ours. They indicated that CIMT was superior to conventional treatment only for improving the quality of life. Although CIMT improved lower limb functions such as movement and balance, the advantage was insignificant compared with conventional treatment. However, their meta-analysis only included six RCTs published in English retrieved from five English language databases. We retrieved publications from four additional Chinese databases to avoid shortcomings due to insufficient sample size and ethnic limitations in individual studies. We conducted the present meta-analysis, which includes 2008 participants from 34 RCTs, to evaluate the effectiveness of CIMT in improving lower extremity motor function in post-stroke patients. We found that CIMT improved lower extremity motor function, balance function, walking speed, mobility, activities of daily living, and quality of daily life more substantially than conventional treatment. Moreover, participants who received CIMT had significantly higher FMA-L scores than those who received only conventional physiotherapy (MD_1_ = 3.46, 95% CI, 2.74–4.17, *P*_1_ < 0.01; MD_2_ = 3.83, 95% CI, 2.89–4.77, *P*_2_ < 0.01) and western medicine (MD_3_ = 3.50, 95% CI, 1.08–5.92, *P*_3_ < 0.01). These results suggest that CIMT is beneficial for patients with motor impairment of the lower limbs after stroke, and secondary indicators show the same trend.

In lower extremity CIMT, the constraint can be behavioral, physical, or both, and aims to improve walking ability and overcome general inactivity. The purpose of lower extremity CIMT is to improve the quality of leg use in the community, but the use of constraint devices remains controversial. For example, dos Anjos (63) noted that using a restraint device would induce yet another type of abnormal coordination pattern. For the legs, a physical restraint could be dangerous and unnecessary. Immobilizing the unimpaired leg (e.g., with knee splints or ankle weight bearing) may alter normal gait or increase inertia in the lower limbs (28, 29). Our results show that lower extremity CIMT with device seemed to yield higher FMA-L scores than lower extremity CIMT without device (with device: MD = 4.52, 95% CI 3.65 to 5.38, *P* < 0.01; with device: MD = 3.37, 95% CI 2.95 to 3.79, *P* < 0.01).

The studies included in this meta-analysis used different CIMT protocol and outcome measures, which may bias of CIMT efficacy assessment. A review (64) mentioned that differences in the types of restrictions, duration of restriction, intervention time, training intensity, and evaluation methods weaken the evidence for the clinical value of CIMT. First, included studies differed in CIMT intensity (ranging from 30 min to 6 h per day) and duration (ranging from 2 weeks to 3 months) because our inclusion criteria did not limit these parameters. One study (21) comparing the effectiveness of two different CIMT protocols pointed out that CIMT intensity can directly affect the patients' recovery time after a stroke. Therefore, it is necessary to develop a unified and effective CIMT protocol according to the different stages of stroke.

Neuroplasticity is typically achieved in response to about 300 highly repetitive tasks per day, typically completed in 1 h (65, 66). However, few of the included studies met that criterion. It is worth noting that, in most included studies, the experimental and control groups performed tasks with similar intensities and durations. Secondly, each study had a specific CIMT scheme. For example, some studies used splints or orthotics to force impaired limb use, some used elevated insoles to force a shift of the weight on the unimpaired lower limb, some used a cane providing auditory feedback to increase the weight on the impaired limb, and some used a 5% weight on the ankle joint. However, using an elevated insole in lower extremity CIMT to force a shift of the gravity center may alter the biomechanics of the lower extremity and impede functional recovery. Therefore, some reviews have opposed the use of shoe raises, advocating that patients should be actively encouraged to maximize the use of impaired limbs (25).

There are three limitations to this study. First, only 4 out of the 34 included studies were rated as “high-quality” through the Cochrane Bias Risk Assessment tool. Therefore, more high-quality studies are needed to provide more reliable evidence. Most researchers did not elaborate on their experimental process, preventing us from accurately judging biases on parameters such as random scheme, blinding method, and allocation hiding. Second, FMA-L, BBS, 10MWT, 6MWT, FAC, TUGT, MBI, and SSQOL, which are important lower extremity motor function indicators, showed pronounced interstudy heterogeneity. Fortunately, removing some studies did reduce heterogeneity. The meta-regression analysis did not identify sample size, publication time, or country as the sources of heterogeneity. As we outlined above, the inclusion criteria did not limit stroke type, duration, affected brain area, hemiplegic side, sample size, or the form, intensity, or duration of CIMT, potentially causing an overestimation or underestimation of CIMT efficacy on lower limb function. Third, to assess publication bias, we performed the Egger's test on more than 10 studies. The test revealed potential publication bias for BBS (P = 0.02) and 10MWT (P = 0.315).

## Conclusion

This study suggests that CIMT improves lower extremity motor function more substantially than traditional physical therapy and western medicine. However, our results should be considered the considerable heterogeneity between studies in mind. Therefore, future studies with larger sample sizes and better quality are needed to demonstrate the benefits of CIMT on lower limb function after a stroke.

## Data availability statement

The original contributions presented in the study are included in the article/Supplementary material, further inquiries can be directed to the corresponding authors.

## Author contributions

MZ, TZ, and HL conceived the study. YT, HL, and TZ provided general guidance to draft the protocol. JW and YT designed the search strategy. MZ drafted the manuscript. MZ, TZ, HL, TY, JC, and PG reviewed and revised the manuscript. All the authors have read and approved the final version of the manuscript.

## Funding

This research was funded by the Xinglin Scholar Research Promotion Project of Chengdu University of TCM (XSGG2019007).

## Conflict of interest

The authors declare that the research was conducted in the absence of any commercial or financial relationships that could be construed as a potential conflict of interest.

## Publisher's note

All claims expressed in this article are solely those of the authors and do not necessarily represent those of their affiliated organizations, or those of the publisher, the editors and the reviewers. Any product that may be evaluated in this article, or claim that may be made by its manufacturer, is not guaranteed or endorsed by the publisher.
