# Supplementary material for: Effect of constraint-induced movement therapy on lower extremity motor dysfunction in post-stroke patients: A systematic review and meta-analysis
Source: Front Neurol. 2022 Nov 21;13:1028206. doi: 10.3389/fneur.2022.1028206 (PMC9720264; doi:10.3389/fneur.2022.1028206)

**Supplemental Figure S1.** the search strategy of WOS


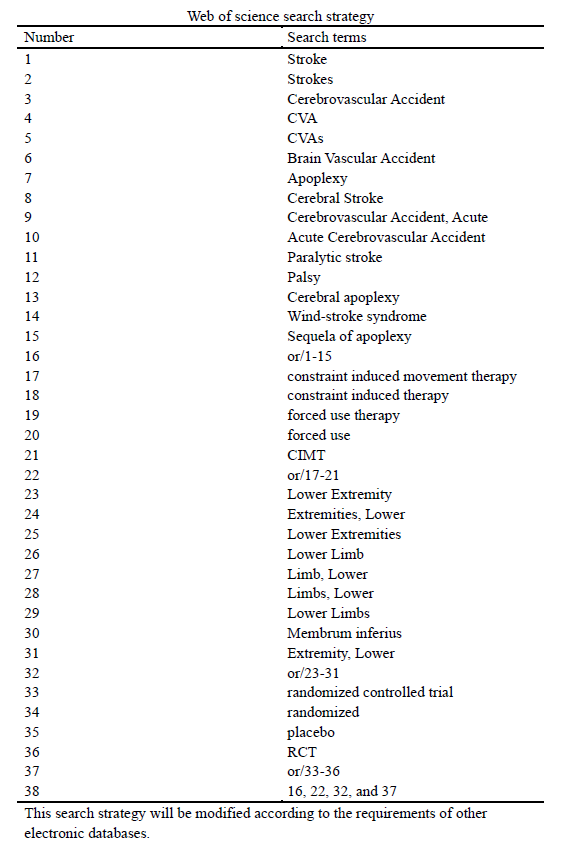


**Supplemental Figure S2.** Risk of bias in the involved studies, assessed by using the Cochrane Collaboration’s risk of bias tool: high risk of bias (+); unclear risk of bias (?); low risk of bias (-).


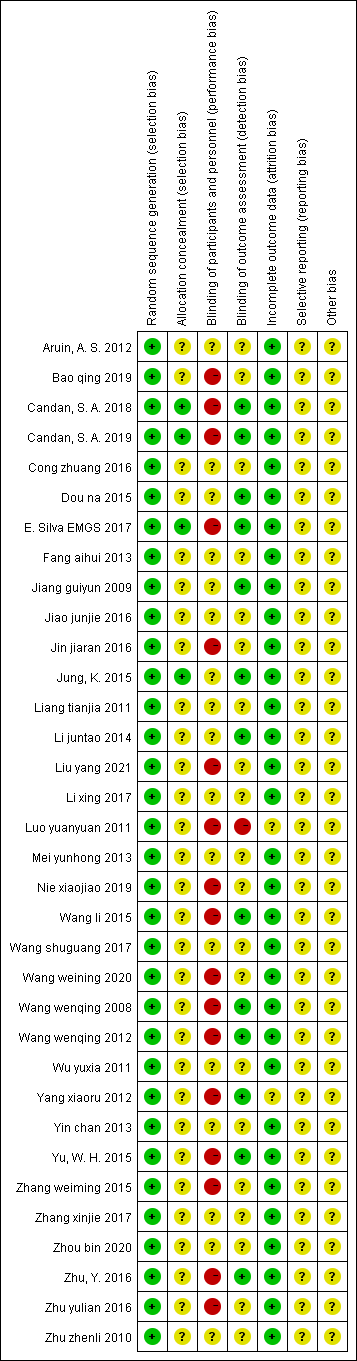


**Supplemental Figure S3.** Graph of the risk of bias: percentage of all studies included.


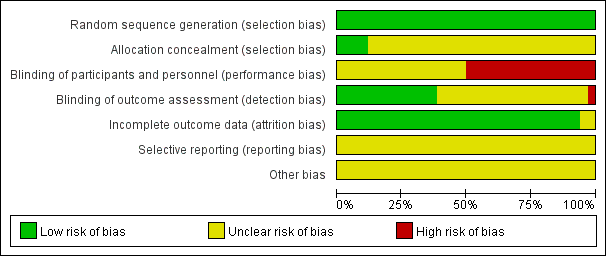


**Supplemental Figure S4.** Funnel plot. CIMT vs Conventional Physiotherapy on the Berg balance scale.


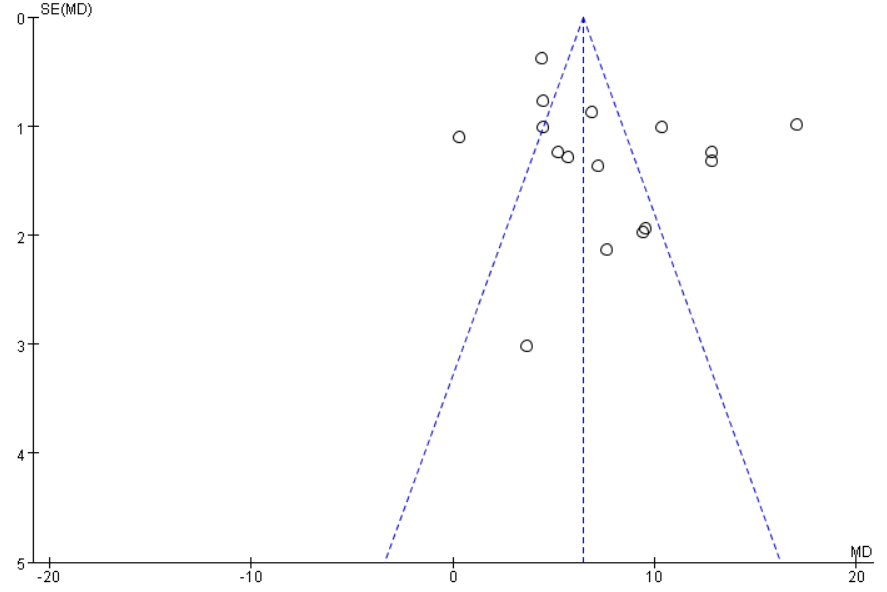


**Supplemental Figure S5.** Egger’s test. CIMT vs Conventional Physiotherapy on the Berg balance scale.


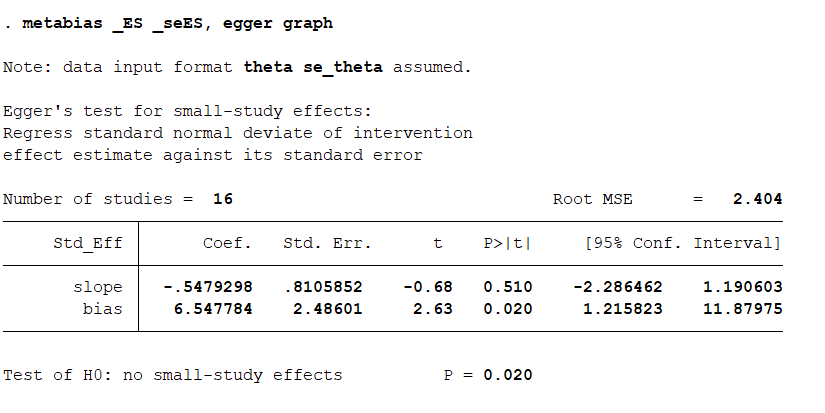


**Supplemental Figure S6.** Funnel plot. CIMT vs Conventional Physiotherapy on the 10-meter walk test.


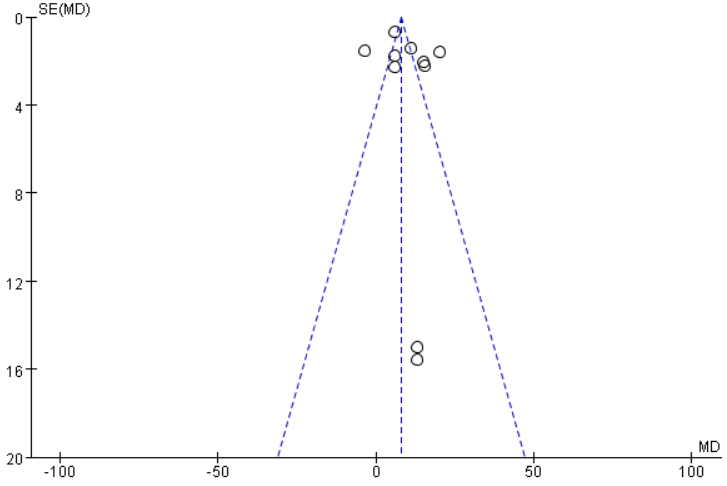


**Supplemental Figure S7.** Egger’s test. CIMT vs Conventional Physiotherapy on the 10-meter walk test.


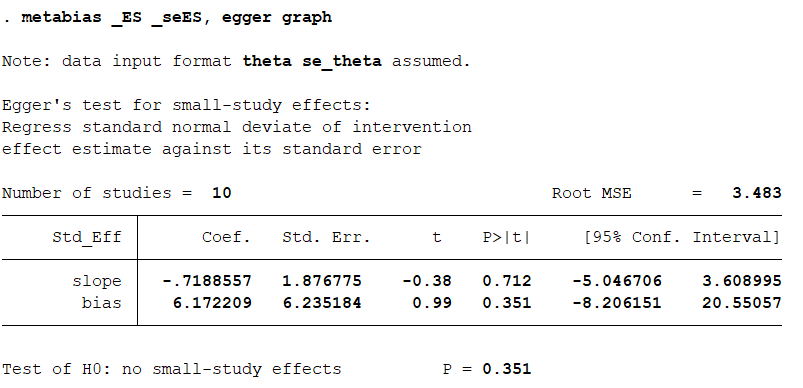

Supplement: Supplementary file 1 [file Data_Sheet_1.docx]
